# Remote Sensing Monitoring of Changes in Soil Salinity: A Case Study in Inner Mongolia, China

**DOI:** 10.3390/s8117035

**Published:** 2008-11-07

**Authors:** Jingwei Wu, Bernard Vincent, Jinzhong Yang, Sami Bouarfa, Alain Vidal

**Affiliations:** 1 State Key Laboratory of Water Resource and Hydropower Engineering Science, Wuhan University, Wuhan 430072, P.R. China. E-Mail: jzyang@whu.edu.cn; 2 Cemagref, BP 44, 92163 Antony Cedex, France. E-Mail: bernard.vincent@cemagref.fr; 3 Cemagref, 361 rue Jean-François Breton, BP 5095, 34196 Montpellier Cedex 5, France. E-Mails: sami.bouarfa@cemagref.fr; alain.vidal@cemagref.fr

**Keywords:** Remote sensing, changing soil salinity, Hetao Irrigation District

## Abstract

This study used archived remote sensing images to depict the history of changes in soil salinity in the Hetao Irrigation District in Inner Mongolia, China, with the purpose of linking these changes with land and water management practices and to draw lessons for salinity control. Most data came from LANDSAT satellite images taken in 1973, 1977, 1988, 1991, 1996, 2001, and 2006. In these years salt-affected areas were detected using a normal supervised classification method. Corresponding cropped areas were detected from NVDI (Normalized Difference Vegetation Index) values using an unsupervised method. Field samples and agricultural statistics were used to estimate the accuracy of the classification. Historical data concerning irrigation/drainage and the groundwater table were used to analyze the relation between changes in soil salinity and land and water management practices. Results showed that: (1) the overall accuracy of remote sensing in detecting soil salinity was 90.2%, and in detecting cropped area, 98%; (2) the installation/innovation of the drainage system did help to control salinity; and (3) a low ratio of cropped land helped control salinity in the Hetao Irrigation District. These findings suggest that remote sensing is a useful tool to detect soil salinity and has potential in evaluating and improving land and water management practices.

## 1. Introduction

Soil salinization is the process of enrichment of a soil in soluble salts that results in the formation of a salt-affected soil [1]. It may occur naturally or as the result of poor management practices. Irrigation modifies the balance of soil hydration by generating an extra supply of water; this supply is always associated with added salts and consequently results in the soil becoming salty and unproductive in the absence of rational management. If the salinity problem cannot be immediately remediated, either for physical, technological, or economic reasons, the land will eventually become totally unproductive and will be abandoned. Consequently, soil salinity seriously threatens the productivity of irrigated land and the livelihoods of the farmers who depend on the affected land [2]. It actually becomes a major threat to sustainable irrigation and thus to food security. It is estimated by IPTRID that 10% to 15% of irrigated areas suffer from varying degrees of salinization, that 0.5% to 1% of irrigated areas are lost each year, and that nearly half of all irrigated areas are threatened in the long-term [1]. The global cost of irrigation-induced salinity is estimated at US$11 billion per year [3].

To combat soil salinization, different technical measures and strategies have been developed in the past century, which can be categorized into physical amelioration, biological amelioration, chemical amelioration, hydrotechnical amelioration and electro-reclamation. However, the success of a reclamation project depends to a large extent on the choice and implementation of the method; it requires a detailed, comprehensive preliminary survey of local conditions and careful definition of the requirements of each reclamation method [4]. However, soil salinity is quite time- and space-dynamic since salinization is the consequence of different complex processes of salt redistribution that depend on natural conditions, system features, agricultural practices and drainage management. In addition, observing the returns and benefits of drainage (the most common salinity control option) takes such a long time (often more than 25 years) that instantaneous measurements of salinity do not reflect current conditions/trends [5, 6]. Observation of many irrigated areas in the world also shows that waterlogging and salinization typically appear only 10 to 50 years after the beginning of the project, depending on the initial depth and recharge rate of the water table and on drainage conditions. Therefore large-scale and multi-temporal studies of salinity, especially of long-term changes in salinity in the past, help to understand the nature of salinization and to evaluate the effectiveness of salinity control practices.

By providing fast, timely, relatively cheap, and repetitive data, remote sensing plays an important role in detecting, mapping, and monitoring salt-affected surface features [**7**]. Large numbers of studies have proven that remote sensing is a useful and promising method to identify salt-affected soils, especially those with high salinity [8-15].

The objectives of this study were: (1) to use archive remote sensing images to depict the history of past spatial and temporal changes in salinity in a large irrigation system; (2) to link this history with land and water management practices and to draw lessons for salinity control. The Hetao Irrigation District (HID), a 2000 year-old irrigation system in North China, was chosen as the study area.

## 2. Study area

**Figure 1.**
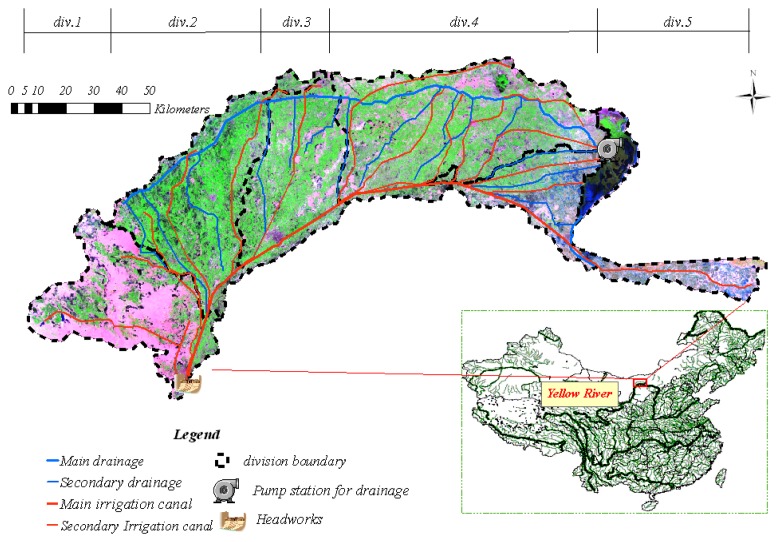
Schematic map of Hetao irrigation District (HID), Inner Mongolia, North China (Based on mosaiced LANDSAT images acquired on August 9, 2001).

The Hetao Irrigation District (hereafter HID) is located in the Inner Mongolia Autonomous region of China, at latitude 40°19′ to 41°18′ and longitude 160°20′ to 190°19′ (Figure 1). It covers a total area of 1,100,000 ha, including 570,000 ha of agricultural land irrigated by water from the Yellow River and is one of the three largest irrigation districts in China. For management requirements, it was divided into five divisions, hereafter named div.1, div.2, div.3, div.4 and div.5, respectively. Physiographically, it is an alluvial river plain with a mean altitude of 1,030 m and a very gentle slope of 1/10,000. However, its microtopography is partly characterized by paleochannels, oxbow lakes, sand dunes, lake marshes and hollows.

The annual average air temperature is 8.1°C, with monthly averages ranging from -10°C in January to 23.7°C in July. Annual potential evaporation can reach 2,200 mm and annual rainfall is about 160 mm on average, 70% of which falls during the monsoon season (July to September). The enormous difference between evaporation and rainfall means that irrigation is absolutely crucial for agriculture, otherwise the soil would be bare. The major source of irrigation water in HID is the Yellow River which has a Total Dissolved Salt (TDS) rate of 0.5 g/L, a sodium adsorption ratio (SAR) of 1.1 and an average pH of 8. The net irrigation depth averages 450 mm/year with a canal conveyance coefficient of 0.47, indicating that 53% of gross inlet irrigation water is "lost" by percolation through unlined irrigation canals. Despite this intensive, long-term irrigation, the groundwater table has remained relatively stable with a mean yearly depth of 1.6 m with variations (0.5 m~3.0 m) related to the seasonality of irrigation.

Soil salinity has always been a threat to the sustainable development of HID, especially after the improvements in the irrigation system and the great expansion of the irrigated area in the 1960s. To combat soil salinity, installation of an artificial drainage system began after 1965, including a drainage canal built in 1965, a pumping station at the outlet built in 1975, and a field drainage system set up between 1989 and 1996, financed by the World Bank. As a result, the annual discharge from the drainage system has increased from 0.15 billion m^3^ to 0.45 billion m^3^ on average, i.e. one-tenth of the total inlet water. The average TDS of the drainage water has increased from less than 1 g/L to 2 g/L, four times that of inlet water. Soil salinity has been gradually controlled. The average grain yield has increased from 1,005 kg/ha before 1960 to 6,450 kg/ha after 1996. It is obvious that the artificial drainage system plays an important role in this achievement. However, on average, 70% of the inlet salt, computed by the administration office using a simple salt balance method, is left inside the system every year. Assuming the salt is distributed evenly throughout the irrigated area, the amount of accumulation would be about 3,100 kg/ha/yr, which could lead to a severe salinity problem.

## 3. Materials and Methods

### 3.1 Data Source

To depict the history of past spatial and temporal changes in soil salinity in HID, archive remote sensing images were used. LANDSAT was chosen as the major source as it is the world's longest continuously acquired collection of space-based land remote sensing data. Similar data from IRS (Indian Remote Sensing satellite) and CBERS (China-Brazil Earth Resources Satellite) were used when needed to compensate for the absence of qualified LANDSAT images.

Images acquired in seven specific years (1973, 1977, 1988, 1991, 1996, 2001, 2006) were selected for this study. For each year, images acquired in March, June and August were used. The images acquired during March, when the land is not yet vegetated and salt surface features are enhanced due to the freeze-thaw process, were used to delineate salt-affected areas. Images acquired in June and August were used to delineate the cropped (irrigated) area, as June corresponds to the mature season for summer crops and August for autumn crops, i.e. when the leaf area reaches its maximum. Images acquired at adjacent dates were used as substitutes if no qualified image was available due to bad weather or sensor failures. Table 1 lists the data used in this study.

To link changes in salinity with land and water management practices, data on annual irrigation depth, annual drainage depth, annual rainfall and monthly averaged groundwater table depth were collected from HID. The groundwater table depth is the average of data from monitoring records of more than 200 wells.

### 3.2 Data preprocessing

All the images used in this study were systematically corrected when purchased. Additional geometric corrections were performed by using ground control points (GCPs) which were collected in the spring of 2004 using a hand-held Garmin GPSmap 76, (Garmin Switzerland). All the images were corrected with an accuracy of less than 0.2 pixels using a second order polynomial transformation. Mercator projection (UTM) Zone 48/49, Spheroid WGS 84 and datum WGS 84 were used as the unified projection parameters. Professional ERDAS IMAGINE 9, (Leica Geosystems LLC), was used to build the mosaics (four LANDSAT MSS scenes were required to cover the zones, while two came from other sensors), to check radiometric calibration, image preprocessing and subsequent classification.

### 3.3 Image classification

The main purpose of image classification was to delineate the salt-affected areas and the cropped areas in different years. To avoid the influence of atmosphere, solar zenith angle and sensors on the reflectance, each image was classified individually based on training samples derived from respective statistics. After achieving satisfactory accuracy for each classification, the two areas and their spatial distribution in the same year were integrated and compared with those in other years.

**Figure 2.**
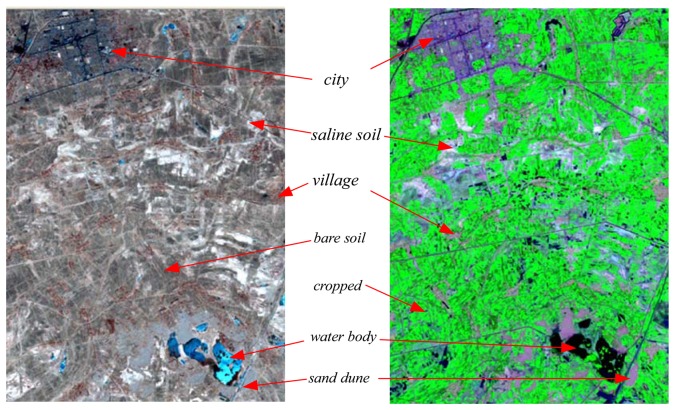
Typical terrain features (Left: 2001-03-11, Band 432; Right: 2001-08-02, Band 7-4-2).

For each classification, the spectral characteristics of different terrain features were first collected to check the possibility of differentiating them from other features. In this study, six major classes were chosen for comparison: vegetation (crops), inhabited areas (villages and towns), saline soils, normal bare soil, water bodies like lakes/ponds, and sand dunes (Figure 2).

**Figure 3.**
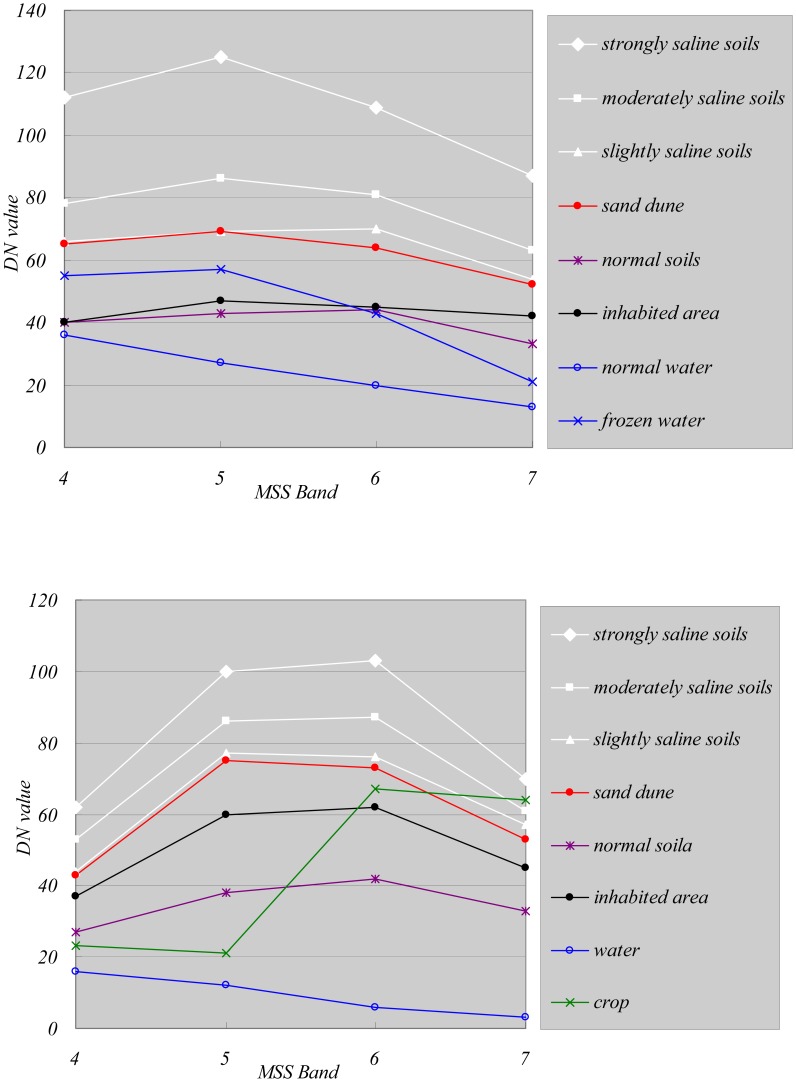
Typical spectral response in Landsat MSS (upper: 1973-03-20, lower: 1977-07-30).

**Figure 4.**
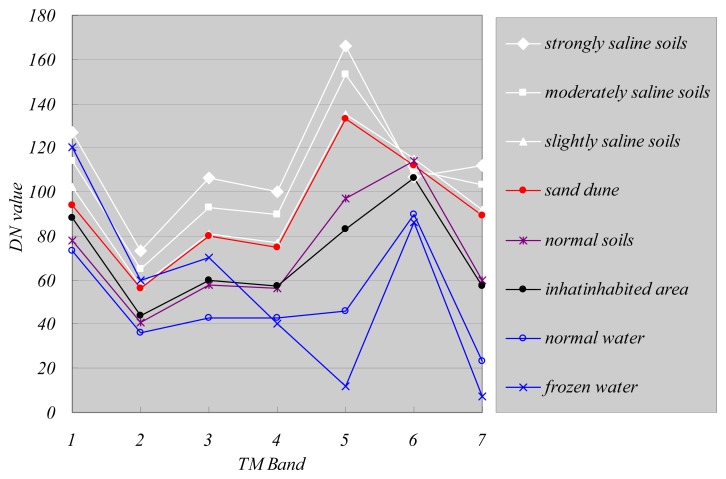

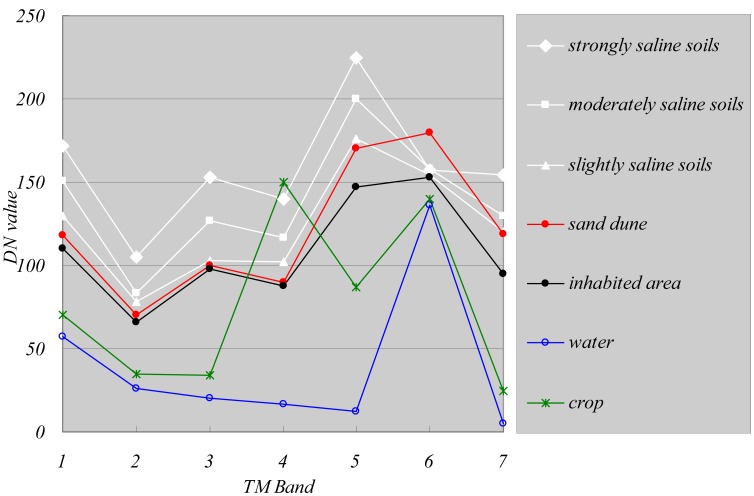
Typical spectral response in Landsat TM (upper: 2001-03-11, lower:2001-08-02).

The vegetated area is bare in the fallow season and is normally irrigated in October to store water for spring wheat. The water bodies are frozen from late October to the following March. The saline soils may be vegetated if the salinity level allows it. Figure 3 and Figure 4 present examples of spectral response patterns for all these classes in both the crop and fallow seasons. These results showed that the spectral response of salt-affected soils, especially strongly and moderately saline soils, is higher than the other classes in all bands and in all images whereas vegetation (mainly crops) displays maximum reflectance in the Landsat TM band 4. The water bodies reflect much less incident energy than other land features, which makes them easy to differentiate. Vegetation has low reflectance in the red band and high reflectance in the near infrared band, which also makes it easy to differentiate it from saline soils. Normal soils, which are generally irrigated and wet, also display lower reflectance than saline soils in all bands, making it possible to differentiate the two. Figures 3 and 4 also show that the difference between slightly saline soils and sand dunes is quite small, especially in MSS images. This can result in major errors when extracting salinity information with the supervised classification method. Fortunately it was possible to minimize this error by using the thermal band in Landsat TM (Band 6). The thermal band is related to the thermal properties of the materials. Sand dunes are much drier than soils and have a lower thermal capacity, so that their temperature tends to be higher in summer (See Figure 4). We were able to take advantage of this thermal characteristic to generate a binary sand dune map from Landsat TM images and then correct the wrongly labeled pixels.

For each year, areas affected by salinity were detected by performing a supervised classification procedure on the image for the period from March to April. This involved supervised training, signature evaluation, selection of band combination and pixel labeling using a maximum likelihood classifier, which are detailed in [16].

Each image was classified into six categories: water bodies, salt-affected areas, sand dunes, inhabited areas, and bare soil. To detect cropped areas, the images acquired during summer/autumn were firstly converted into Normalized Difference Vegetation Index (NDVI) images using the band ratioing technique and then classified into 256 classes using an unsupervised classification method. This is based on the fact that the limit between irrigated and non-irrigated areas is metric and is as contrasted as bare soil vs. vegetated areas.

**Figure 5.**
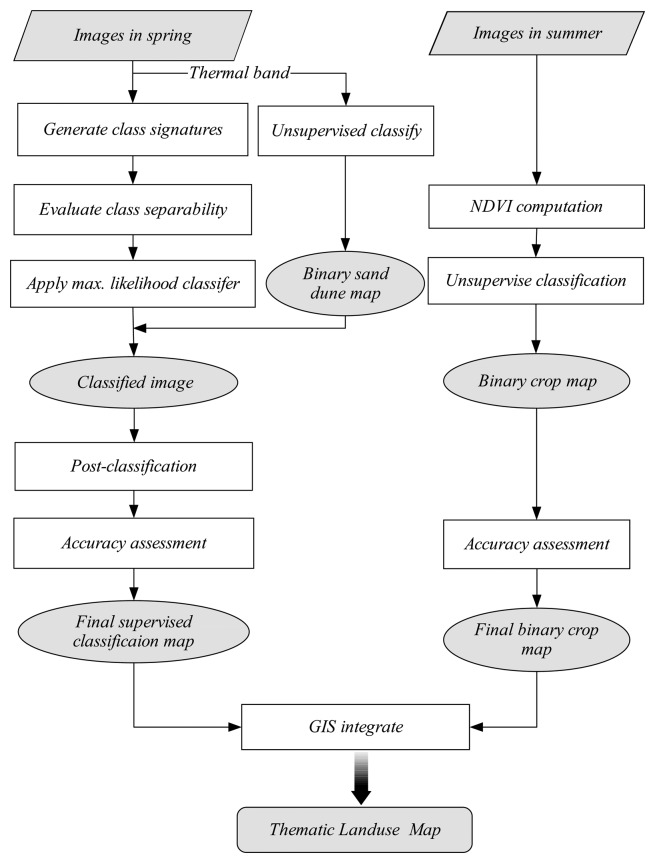
Procedure of image classification and land use mapping.

By overlaying these classified images with the corresponding original images using swipe utility, and changing the threshold values, they were finally recorded into binary images (cropped or non-cropped). By adding the binary images in the summer to those acquired in the same autumn, images of the cropped area for the respective year were acquired. Next the classified images acquired during spring (i.e. salt-affected areas) and the images of the cropped area were integrated into one thematic land use map for the corresponding year using the GIS module in ERDAS. In each thematic land use map, nine categories of land use were indexed, i.e. salt-affected bare soil, bare soil, sand dunes, villages, towns, two classes of surface water bodies (one reedy, one reed-free.) salt-affected areas, and salt-free cropped areas. The above procedure is summarized in Figure 5.

### 3.3 Ground verfication data collection

Field investigation and sampling were conducted on May 15, 2006 before irrigation to evaluate the accuracy of salinity detection using remote sensing. Initially, a reconnaissance of the area was made to identify and locate typical surface features. Most points were located in abandoned land areas where serious salinity tends to occur. Then a total of 153 soil samples were taken from the 0-5 cm surface soil layer and located using the same GPS as the one used for collecting GCPs. The total salt content of the soil was measured according to Chinese standard laboratory procedures and then graded into four categories: slightly saline, moderately saline, highly saline and solonchak (very highly saline) according to the soil classification method used by HID technical services and also by other related Chinese institutes [17, 18]. The results were then used for the one-to-one check with the results of corresponding classification.

Agricultural statistical data were also collected from the local government to evaluate the accuracy of the detection of cropped areas using remote sensing. The cropped areas were compared with those from the corresponding classification division by division for the corresponding year.

## 4. Results and Discussion

### 4.1. Crop and salinity maps

Following the above method, land use maps were built for the seven years concerned, (these are available in electronic format as supplementary material). The GIS analysis function of ERDAS software was used to identify the cropped areas, salt-affected areas and their spatial relations. Several percentages were also derived, including (1) the percentage of salinity in the cropped area (=salt-affected cropped area/total salt-affected area); (2) the percentage of the salt-affected cropped area (= salt-affected cropped area/total cropped area) and (3) the percentage of cropped area (= total cropped area/system command area). These data are plotted in the following figures.

### 4.2. Accuracy assessment

During the supervised classification of salt-affected areas, producer accuracies, user accuracies and overall accuracies were calculated with error matrixes (not presented here) for all available images. The KHATs, values derived from the kappa coefficients of agreement, which are the measurement of chance agreement [19], were also calculated to evaluate the quality of each classification. These accuracies and KHATs show that the classification accuracies are acceptable. Using the image acquired on March 11, 2001 as an example, 95.5% of the image area was correctly classified. The producer's accuracy of salt-affected soil was 93.3%, indicating the producer of classifier correctly classified the proportion of saline soil pixels. The user's accuracy of salt-affected soil was 85.7% indicating that 85.7% of the pixels labeled as salt-affected soil on the classified image were actually salt-affected soil. The classified image produced a KHAT of 0.947 indicating that the classification process avoided 94.7 percent of the errors that a completely random classification would generate. Similar accuracies were achieved for other images.

The one-to-one check showed that overall accuracy was 90.2%, indicating that the classification was satisfactory (Table 2). The check also showed that the accuracy of salinity detection using remote sensing increased with the level of salinity. The slightly saline soil was the least accurate (78.9%) while the solonchak was the most accurate (93.8%). Hence remote sensing proved to be efficient in detecting salinity in HID.

**Figure 6.**
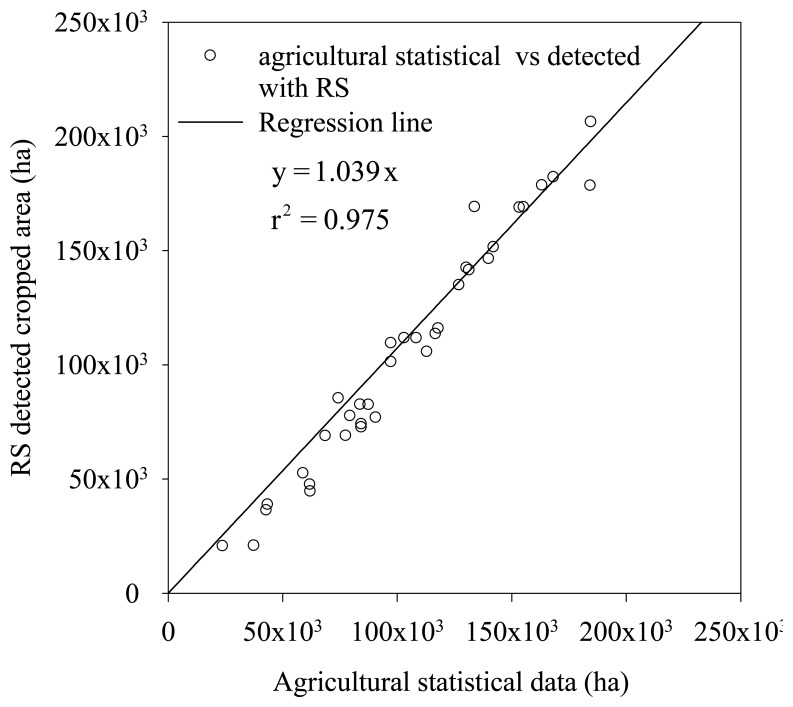
Validation of cropped area using agricultural statistical data.

Figure 6 plots the cropped areas detected by remote sensing vs. those derived from the agricultural statistics for the different divisions supplied by the administration office of HID. It shows a good agreement between the cropped areas from two sources. Calculation shows that the average difference was about 2%.

### 4.3. Changes in salinity and water management

**Figure 7.**
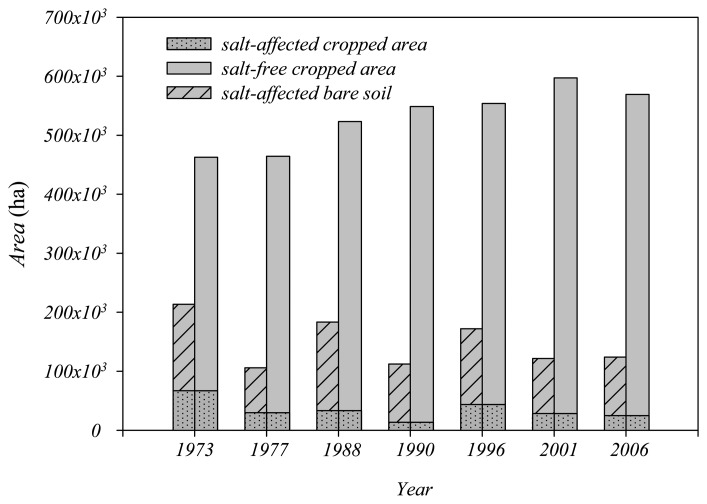
Salt-affected area, bare soil and salt-free cropped area in HID.

The cropped areas and salt-affected areas detected by remote sensing for the years 1973, 1977, 1988, 1990, 1996, 2001, and 2006 are presented in Figure 7. The cropped area includes two parts: salt-affected cropped areas and salt-free cropped areas. The total salt-affected area also includes two parts: salt-affected cropped areas and salt-affected fallow areas.

The cropped area increased from 453,000 ha in 1973 to 596,000 ha in 2001 and decreased slightly from 2001 to 2006. On the other hand, the total salt-affected area decreased from 213,500 in 1973 to 124,000 in 2006. The corresponding ratio of salt-affected area to system command area decreased from 19% (=213,500/1,100,000) to 11% (=124,000/1,100,000). The salt-affected cropped area also decreased from 67,100 ha in 1973 to 24,900 ha in 2006. In spite of two rebounds in 1988 and 1996, the general extent of salinity in HID remained relatively stable. Moreover, the salt-affected cropped area decreased from 43,800 ha in 1996 to 28,400 ha in 2001, while the corresponding cropped area increased from 554,000 ha to 597,000 ha. This decrease in salinity indicates that the installation of a pumping station for drainage in 1975 and the improvement of the field drainage system completed in 1996 had obvious positive effects on salinity control.

### 4.4. Changes in salinity and land management

For an irrigation system to be successful and sustainable, it is widely accepted that a natural or artificial drainage system must be available to remove excess water and salt from the irrigated soils. In many instances, the natural system has been found to be inadequate, and providing conventional drainage in the form of surface and subsurface drainage has proven to be very costly. To secure land from salinity hazard, an alternative method named “dry drainage” was proposed for salinity control[20]. In this method, part of the irrigated land is set aside and acts as a sink for excessive irrigation water and for the salt transported with the groundwater. The land is left fallow. The natural groundwater system then provides the pathway for the movement of the excessive irrigation water from the irrigated/cropped area to the fallow area [20]. This method has been tested with model calculations and field experiments and proved to be useful for salinity control [20-24]. However tests at the scale of the whole system and long-term tests are needed to validate its effectiveness and sustainability.

**Figure 8.**
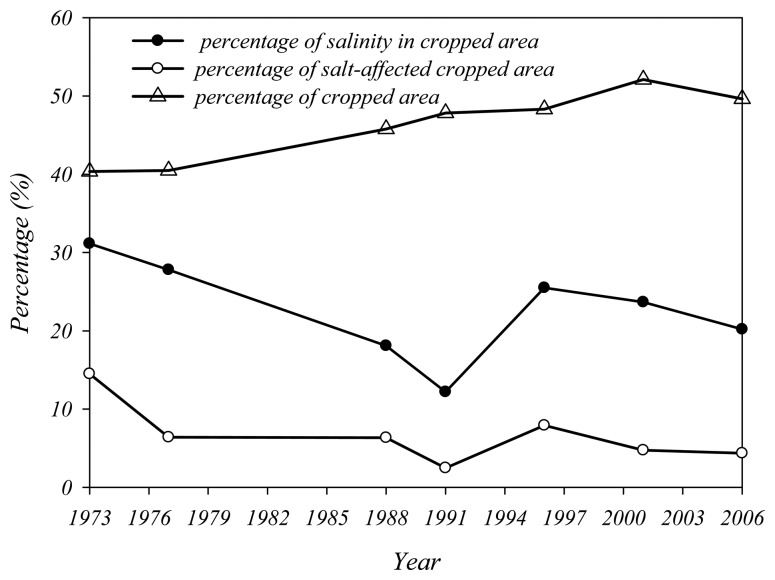
The percentages of cropped area, salinity in cropped areas and salt-affected cropped areas.

**Figure 9.**
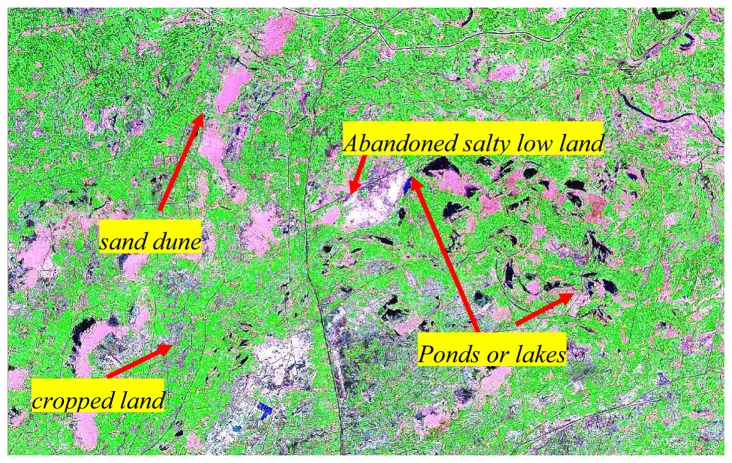
General land cover in HID (snapshot of LANDSAT image P129 r31 on August 9, 2001 at a scale of 1:150,000).

In HID, as described above, around 70% of the inlet salt is left inside the system every year. However the percentage of salt-affected cropped area ranged from 2.5% to 14.5% with an average of 6.6% (the line with open circle symbols in Figure 8) in the last 34 years. This success is attributed to both the artificial drainage system and the dry drainage. The percentage of cropped area (the line with triangle symbols in Figure 8) ranged from 40% to 52%, meaning that half of the land was available to drain excess water and salt when topographic and geo-hydrological conditions allowed it. Figure 9, a snapshot of a LANDSAT image acquired on August 9, 2001, shows the general land cover in HID. The cropped area is surrounded by abandoned sand dunes, ponds, lakes, and salty low land that form the natural outlet for salted groundwater. This practice ensures that salt is drained laterally from the cropped area to avoid serious salinity hazard, even in the absence of artificial drainage. For instance, the salt-affected cropped area represented only 14.5% in 1973 when there was no effective artificial drainage (Figure 8). Additionally, the percentage of salinity in the cropped area (the line with filled circles in Figure 8) remained lower than 30%, meaning that salinity mainly occurred in the fallow area, where salts continuously accumulated.

Figures 7, 8 and 9 show that dry drainage plays an important role in maintaining salinity in the cropped area at an acceptable level. It can be used as an effective alternative method for salinity control when artificial drainage is not feasible or affordable. For the land manager, it may be more satisfactory to set aside part of the land as fallow rather than maximize land cropping. To complete this analysis, it would be necessary to conduct a comparative cost-benefit analysis of dry drainage, and conventional drainage, vs. agricultural product loss due to soil salinity.

## 5. Conclusions

In this study, archive images were used to depict the history of changes in soil salinity in Hetao Irrigation District, and field sampling data and agricultural statistical data were used to check accuracy. Results showed that the overall accuracy of remote sensing in detecting soil salinity was 90.2%, and 98% in detecting cropped area, which suggests remote sensing is a useful tool to detect soil salinity and to monitor changes in soil salinity.

The historical irrigation/drainage data were used to analyze the relationship between changes in soil salinity and land and water management practices. Several important findings were that (1) setting up an artificial drainage system did help control salinity; and (2) maintaining a high ratio of fallow land to cropped land helped maintain a dry drainage flux that effectively helped HID to control salinity. These findings suggest that remote sensing has potential in evaluating and improving land and water management practices.

## Figures and Tables

**Table 1.** The images used in this study.

**Data source (Sensor)**	**Images for salinity detection**	**Images for cropped area detection**
LANDSAT (MSS)		
Green (Band 4): 0.50-0.60 μm	1973/12/14 P138/R31,32	1977/06/05 P138/R31,32
Red (Band 5): 0.60-0.70 μm	1973/03/20 P139/R31,32	1977/07/30 P139/R32
Near IR (Band 6): 0.70-0.80 μm	1977/04/12 P138/R31,32	1979/10/09 P139/R31
Near IR (Band 7): 0.80-1.20 μm	1977/04/13 P139/R31,32	1977/08/16 P138/R31,32
LANDSAT TM		
Blue (Band 1): 0.45-0.52 μm	1988/04/08 P128/R31	1988/08/14 P128/R31; 1988/07/04 P129/R31
Green (Band 2): 0.52-0.60 μm	1988/04/15 P129/R31	1991/06/20 P128/R31; 1991/06/27 P129/R31
Red (Band 3): 0.63-0.69 μm	1990/03/29 P128/R31	1991/08/07 P128/R31; 1991/08/30 P129/R31
Near IR (Band 4): 0.76-0.90 μm	1990/03/04 P128/R31	1996/06/17 P128/R31; 1996/06/24 P129/R31
Mid IR (Band 5): 1.55-1.75 μm	1996/03/13 P128/R31	1996/09/05 P128/R31; 1996/08/27 P129/R31
Thermal (Band 6): 10.4-12.5 μm	1996/04/21 P129/R31	2001/07/01 P128/R31; 2001/06/22 P129/R31
Mid IR (Band 7): 2.08-2.35 μm	2001/03/11 P128/R31	2001/08/02 P128/R31; 2001/08/09 P129/R31
	2001/03/18 P129/R31	
IRS (AWIFS)		
Green 0.52-0.59 μm		
Red 0.62-0.68 μm	2006/03/28 P124/R40	
Near Infrared 0.77−0.86 μm		
Short wave infrared 1.5-1.7 μm		
CBERS (CCD)		
Pan band 0.51-0.73 μm		2006/06/16 P11/R54,55; 2005/06/25 P10/R54 2004/07/20 P9/R54
Blue 0.45-05.2 μm		
Green 0.52-0.59 μm		
Red 0.63-0.69 μm		
Near infrared 0.77-0.89 μm		

**Table 2.** Accuracy assessment using field sampling data.

**Salinity degree**	**Salt content (g/100g)**	**No of samples**	**No of correct detections**	**Accuracy**
Slightly saline	0.2-0.3	18	14	78.9%
Moderately saline	0.3-0.6	18	15	83.3%
Strongly saline	0.6-1.0	20	18	90.0%
Solonchak	>1	97	91	93.8%

Overall		153	138	90.2%

## References

[1] CISEAU (2006). Extent of Salinization and Strategies for Salt-affected Land Prevention and Rehabilitation:Background Paper. Electronic Conference on Salinization organised and coordinated by IPTRID.

[2] Umali D.L. (1993). Irrigation-Induced Salinity: A Growing Problem for Development and the Environment.

[3] Food and Agriculture Organization of the United Nations (2005). Management of irrigation-induced saltaffected soils..

[4] Food and Agriculture Organization of the United Nations (1973). Irrigation, Drainage and Salinity: an international source book..

[5] Jury W.A. (1975). Solute travel-time estimates for tile-drained fields: I. Theory. Soil Sci. Soc. Am. J.

[6] Jury W.A. (1975). Solute travel-time estimates for tile-drained field: II. Application to experimental studies. Soil Sci. Soc. Am. J.

[7] Metternicht G.I., Zinck J.A. (2003). Remote sensing of soil salinity: potentials and constraints. Remote Sens. Environ..

[8] Mohammed Saifeldeen A.E. (2005). Assessment of soil salinity problems in agricultural areas through spatial and temporal remote sensing. Ph.D.dissertation.

[9] Rao B.R.M., Sankar T.R., Dwivedi R.S., Thammappa S.S., Venkataratnam L., Sharma R.C., Das S.N. (1995). Spectral Behavior of Salt-Affected Soils. Int. J. Remote Sens..

[10] Metternicht G.I., Zinck J.A. (1996). Modelling salinity-alkalinity classes for mapping salt-affected topsoils in the semiarid valleys of Cochabamba (Bolivia). ITC J..

[11] Dehaan R., Taylor G.R. (2003). Image-derived spectral endmembers as indicators of salinisation. Int. J. Remote Sens..

[12] Vincent B., Vidal A., Tabbet D., Baqi A., Kuper M. (1996). Use of satellite remote sensing for the assessment of waterlogging and salinization as an indicator of the performance of drained system. 16th International Congress on Irrigation and Drainage, Proc. Workshop on the Evaluation of Performance of Subsurface Drainage Systems.

[13] Vidal A., Tabet D., Ahmad M. D., Asif S., Zimmer D., Strosser P. (1998). Salinity assessment in irrigation systems using remote sensing and geographical information systems - application to Chistian subdivison, Pakistan.

[14] Tabet D. (1999). Intérêt d'une approche spatiale pour suivi de la salinité des sols dans les systèmes irrigués: cas de la subdivision de Chistian dans le Punjab, Pakistan. Ph.D. dissertation.

[15] Vincent B., Pereira L.S., Cai L.G., Musy A., Minhas P.S. (2003). Remote sensing for spatial analysis of irrigated areas. Water savings in the Yellow River Basin:Issues and decision support tools in irrigation.

[16] Geosystems L. (2005). ERDAS IMAGINE Tour Guide.

[17] (1981). Nanjin Institute of Soil Science. Physico-chemical Analysis of Soil..

[18] Wang Z. (1993). Saline Soil in China..

[19] Congalton R.G., Green K. (1999). Assessing the accuracy of remotely sensed data: principles and practices..

[20] Khouri N. (1998). Potential of dry drainage for controlling soil salinity. Can J Civil Eng.

[21] Greenwood E.A.N., Milligan A., Biddiscombe E.F., Rogers A.L., Beresford J.D., Watson G.D., Wright K.D. (1992). Hydrologic and Salinity Changes Associated with Tree Plantations in a Saline Agricultural Catchment in Southwestern Australia. Agr. Water Manage..

[22] Xiao M. (1994). Entironment and reclamation of the saline-sodic lands in the oasis of Tianshan Mountain. Environ. Prot. Xinjiang.

[23] Gowing J.W., Wyseure G.C.L. (1992). Dry-drainage a sustainable and cost-effectibe solution to water logging and salinisation.

[24] Konukcu F., Gowing J.W., Rose D.A. (2006). Dry drainage: A sustainable solution to waterlogging and salinity problems in irrigation areas?. Agr. Water Manage..

